# Supplementary material for: Cognitive performance outcomes: considerations for drug development
Source: J Patient Rep Outcomes. 2023 Oct 19;7:102. doi: 10.1186/s41687-023-00644-1 (PMC10587033; doi:10.1186/s41687-023-00644-1)
Supplement: Supplementary file 1 — Supplementary Material 1 [file 41687_2023_644_MOESM1_ESM.docx]

**Abstract**

Ensuring that cognitive assessments are fit for purpose is critical in the evaluation of the clinical benefit of new therapeutic interventions. Although guidelines for Clinical Outcome Assessments (COA) are available, performance outcome (PerfO) assessments, and in particular those assessing cognition (Cog-PerfOs) are more complex and have additional requirements that need to be considered. I outline three areas where further discussion around validation methods for Cog-PerfOs and best practices is warranted: 1) content validity 2) ecological validity, and 3) construct validity in multinational contexts. I conclude with a discussion of several potential avenues for the improvement of validation of Cog-PerfOs used to evaluate the efficiency of medical products that target cognitive symptomatology. These include the involvement of cognitive psychologists in establishing content validity of Cog-PerfOs, evaluating the congruence of laypeople’s and expert understanding of cognitive concepts, supplementing qualitative with quantitative evidence when establishing content validity, demonstrating ecological validity, and ensuring normative data are available in multinational contexts.

**Introduction**

Cognitive impairment implies difficulties in thinking, learning, concentrating, remembering, and making decisions that affect everyday life. In many neurology and psychiatry areas where impaired cognition constitutes key symptomatology, such as Alzheimer’s disease (AD) and age-related dementia, Parkinson’s disease dementia, or schizophrenia, there are few, if any, approved therapies targeting these symptoms. A common feature of clinical research in these indications is that it relies on cognitive performance outcome assessments (Cog-PerfO) - a type of Clinical Outcome Assessment (COA) as a primary and/or key secondary outcome. Cog-PerfOs are measurements of mental performance completed through answering questions or performing tasks.

There has been a relative lack of guidance on PerfOs, which is increasingly being recognized by academia, industry, and regulators. Recent work has started to focus on initiating the discussion and providing some recommendations for their use[1,2]. Due to the nature of cognitive phenomena, there are even more specific challenges when it comes to Cog-PerfOs than for other COAs in demonstrating their validity in the context of drug development. This commentary will outline three areas where further discussion around validation methods is warranted and suggest ways to address them.

**What are some of the challenges of Cog-PerfOs in drug development?**

1. **Content validity**

Content validity relates to the extent a COA represents the concept of interest, that the items and domains are appropriate and comprehensive with regards to its intended measurement concept, and context of use (COU) [3]. It is a prerequisite for other types of validity and as such is of the highest priority during COA development. An absence of content validity would mean that the COA fails to comprehensively and/or accurately cover the relevant symptoms associated with the condition, which would lead to faulty assessments. For example, when assessing AD, to encompass the full scope of cognitive deficits associated with the disease, a Cog-PerfO battery would need to cover a range of cognitive functions, such as memory, language, executive functions, spatial awareness, rather than solely focusing on, for instance, memory. For each of these functions, the Cog-PerfO domain(s) would need to be appropriate for the COU. If the targeted population are individuals in the early stages of AD, their primary language difficulty is likely to revolve around difficulties in recalling words, as opposed to the broader language challenges seen in more advanced stages of the condition, such as comprehension and spontaneous speech deficits. Therefore, in demonstrating content validity, patient and/or caregiver input is required so that the concepts relating to the feelings, functioning, and activities adversely affected by the disease are appropriately captured. To demonstrate a treatment benefit, COAs need to represent a meaningful aspect of health, i.e., one that the patient cares to improve.

There has been much debate on how to support the content validity of Cog-PerfOs and whether it is possible to apply a qualitative approach [4] or to use quantitative approaches[5]. This is because, unlike other concepts that reflect how the patient feels and functions, cognitive concepts are less clear to define and report. There are more variations in the *conceptualization* of cognitive abilities than is the case for other COA constructs*.* For many concepts such as attention, memory, intelligence, or executive functions, there is no universally accepted definition. Some cognitive concepts, such as executive functions, are less obvious to recognize and report than abilities that are more easily observed such as memory or language. Cognitive concepts are often “umbrella terms” encompassing a set of related but diverse functions.

Despite the aforementioned issues, the expectation is that patients can easily report all cognitive symptoms and that experts and patients share the same or similar understanding of the concepts. However, a study comparing laypeople's and experts’ understanding of concepts of cognition for 18 neuropsychological tests, suggested that the domain of attention has discordance, while concepts such as language, memory, and executive functions/thinking have concordance between experts and laypeople[6]. This indicated that the conceptual understanding of these terms between experts and patients might not be always aligned which can interfere with content validation and task selection.

1. **Ecological validity**

Ecological validity refers to the extent an assessment is congruent with real-world functioning. Cognition impacts day-to-day life and functionality, however, many Cog-PerfOs are derived from laboratory experiments in psychology. Hence, their ecological validity and demonstrating how they relate to the feelings and functioning of patients is not self-evident. A memory task where participants are required to recall a list of random words that were presented for a short period on a computer screen would be useful for studying certain aspects of memory, and even for identifying impairment compared to a norm. However, it would fully not represent how people remember information in their daily lives, where information is often remembered in context. Memory challenges of patients with AD, for instance, might include forgetting names, getting lost, or struggling to follow conversations, which are not assessed by these tasks.

While we can hypothesize that Cog-PerfOs do have a role in meaningful functional activities, without establishing ecological validity, the meaning of these scores cannot be determined. Cog-PerfOs are often not tested in terms of their correlates with concerns in daily life, including those frequently used such as the ADAS-Cog[4].

1. **Cog-PerfOs in Multinational Contexts**

Cog-PerfO scores can be affected by different cultural contexts in additional ways than other COAs that have a questionnaire format, such as patient-reported outcomes, observer-reported outcomes, and clinician-reported outcomes. Most Cog-PerfOs have been developed in Western cultures and some items and processes may not be contextually appropriate in another setting. For example, stimuli used in a Cog-PerfO might not be familiar in certain cultures which can impact scores of some cognitive constructs, such as working memory capacity[7]. Similarly, as cognition is shaped by the environment[8], standard cognitive tests using numerical concepts or abstract thinking are influenced by formal education and may not adequately reflect cognition in certain cultures. For example, remote Aboriginal people underperform on standard cognitive tests[9] but show superior visuospatial abilities in comparison to non-Aboriginal Australians as a useful skill for desert-way finding[10].

Another aspect that is important to consider when using Cog-PerfOs in a multinational context is the availability of normative data. Normative data serve as a reference as to what would be typically expected and are based on diversity characteristics such as age, gender, education, ethnicity, and socioeconomic status. Normative data are important for guiding interpretation, understanding the severity of issues versus a control population, and enabling comparison between cognitive domains and different geographical locations. Derivation and interpretation of thresholds of meaningful scores for PerfOs are challenging and norms are useful in this regard as a distribution-based estimate. Normative data from one population cannot be applied to another, so they need to be available for all populations participating in a trial. The time of test standardization (i.e., when the norms were created) should also be considered in multinational trials. A phenomenon called “the Flynn effect” suggests that there is a substantial increase in cognitive test scores over time[11]. If norms have been created at different time points in various countries, there can be inflation of scores resulting in overestimated outcomes. This may be particularly true in countries with fewer resources where norms may not be up to date.

**Where do we go from here?**

Consensus building is necessary to develop guidelines and good practices for Cog-PerfOs in drug development. To inform the progress of these debates, researchers can pursue several evidence-building approaches to improve demonstrating the validity of Cog-PerfOs.

1. **Involve cognitive psychologists in content validation and task selection**

Including cognitive psychologists in the content validation of Cog-PerfOs is essential. Cognitive psychologists can take part in concept elicitation activities so that concepts are appropriately identified, thereby improving task selection and/or adaptation. For example, in a concept elicitation interview, a patient might bring up “memory problems” and the discussion guide, interviewer, or coder would need to know how to gather information and/or distinguish the appropriate memory function, such as episodic or working memory. Cognitive psychologists could provide their input by putting together interview guides, training interviewers about the meaning of cognitive concepts, and through consensus panels (e.g., Delphi methods) where results can be reviewed and shared for interpretation, to assure that patient input has been mapped to the appropriate cognitive constructs and through selecting tasks that match the elicited concepts. The involvement of experts may also help in minimizing COA administration burden by selecting more appropriate cognitive tasks rather than a broad variety of tasks.

In multinational trials, cultural adaptations of Cog-PerfOs might be necessary. While cultural adaptations can increase validity in a certain context, such changes can also reduce the comparability between the source test and the adapted version, which can impact content validity or measurement properties between the versions used. Cognitive psychologists can inform the extent to which the cultural context can impact the construct being measured[7]. The Cog-PerfO might need different versions of the tasks and stimulus materials designed for use in different countries, in addition to modifications of the language of the instructions.

1. **Explore concepts in lay language and tasks**

An interesting approach to addressing the imprecision of cognitive concepts between expert use and everyday language is to collect evidence on how laypeople (i.e. patients) map the selected Cog-PerfOs to one or more predefined cognitive domains[6]. This could serve as a simple approach to support the conceptual relevance of the selected tasks and useful supporting evidence for Cog-PerfO content validation. It might be useful to also see whether the understanding of these concepts varies by language/country. However, while congruence is useful evidence, incongruence might not necessarily mean that the tasks are inadequate for the purpose – but is nonetheless informative of possible conceptual misunderstandings between laypeople and experts that should be considered.

1. **Support content validity with quantitative data**

While demonstrating content validity for other COAs is more straightforward with qualitative methods, evidence for Cog-PerfOs would benefit from adding quantitative evidence to the qualitative data[1]. Such additional quantitative evidence could unearth early, subtle cognitive deficits, especially for conditions such as mild cognitive impairment that cannot be observed via self-report.

1. **Demonstrate the ecological validity of cognitive tasks**

Ecological validity of cognitive tasks can be evaluated via two approaches: a) generalizability - the extent to which Cog-PerfOs predict behaviors outside of the test environment, i.e. functional outcomes, and/or b) representativeness – the extent to which Cog-PerfOs resemble real-life contexts [12]. Establishing ecological validity through generalizability is done by correlating Cog-PerfOs with measures of everyday functioning [12]. This can be an important first step toward establishing which instruments have more potential for demonstrating clinical meaningfulness. However, it remains to be seen which standard measures can satisfy this criterion as most have not been developed with ecological goals in mind[12]. The representativeness approach may facilitate demonstrating that a Cog-PerfO has relevance and purpose to the patient, but this would largely require abandoning existing tests and developing new ones[12]. However, some recent efforts in the direction of representativeness have included the development of virtual reality Cog-PerfOs, such as the Virtual Reality Functional Capacity Assessment Tool (VRFCAT)[13], which has been accepted into the US Food and Drug Administration’s (FDA) COA qualification program. Here, users navigate their way through a series of real-world tasks associated with cognitive functioning, such as cooking, grocery shopping, or taking transport. Methods for determining ecological validity – whether using generalizability, representativeness, or both – need to be evaluated depending on the specific Cog-PerfO at hand, the subject population, and the goals of the project.

1. **Ensure appropriate normative data are available for diverse populations**

Norm-based standardized scores based on normative data with patients and healthy comparison subjects matched to the census of a certain country on age, education, race, and sex should be available. This is especially important if the patient sample in a clinical trial is diverse in a way that could impact the cognitive construct beyond individual differences, and hinder interpretation. Published data could inform whether the construct varies depending on certain characteristics such as age or culture. Many Cog-PerfOs will have some normative data published in manuals – at least for some geographical locations. A decision tree regarding the need for norms is shown in Figure 1. **
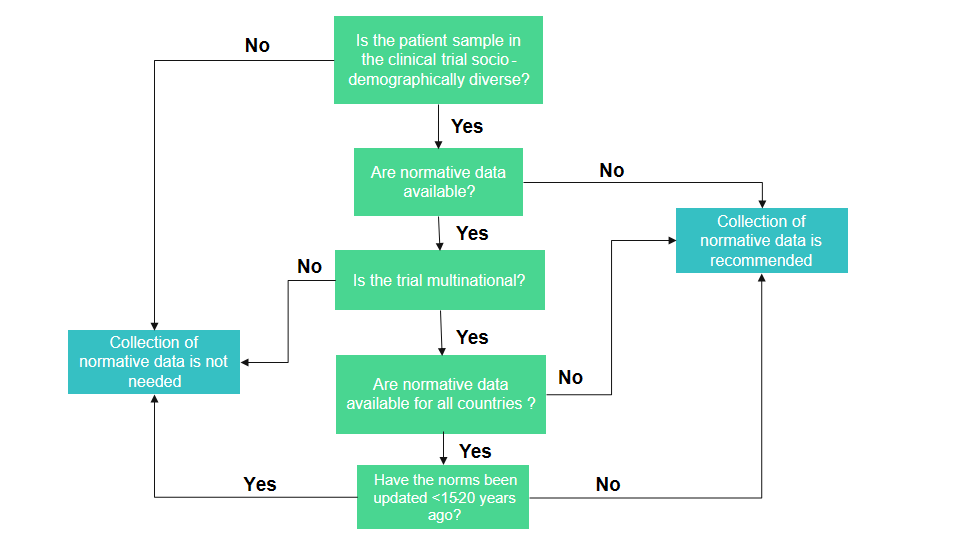
**

**Figure 1.** Decision tree regarding the need to collect normative data for a Cog-PerfO.

**Concluding remarks**

A COA is considered fit-for-purpose when its validation supports its COU. Adjusting standard operating procedures when using Cog-PerfOs in drug development to include cognitive psychologists in the development and adaptation of Cog-PerfOs, evaluating the congruence of laypeople’s and expert understanding of cognitive concepts, supporting qualitative with quantitative evidence in content validity, and ensuring normative data are available to account for population differences over time and geography. While this endeavor may seem effort-intensive, such work would lead to the appropriate development, selection, and adaptation of Cog-PerfOs in certain disease contexts, so that valid and sensitive tools are available to determine if a medical product can delay disease progression or improve cognition.

**References**

[1] Edgar CJ, Bush E (Nicki), Adams HR, et al. Recommendations on the Selection, Development, and Modification of Performance Outcome Assessments: A Good Practices Report of an ISPOR Task Force. Value in Health. 2023;26:959–967.

[2] Richardson E, Burnell J, Adams HR, et al. Developing and Implementing Performance Outcome Assessments: Evidentiary, Methodologic, and Operational Considerations. Drug Inf J. 2019;53:146–153.

[3] US Food and Drug Administration. Guidance for Industry—Patient-Reported Outcome Measures: Use in Medical Product Development to Support Labeling Claims. 2009. [Internet]. Available from: www.fda.gov/media/77832/download.

[4] Ropacki MT, Hannesdottir K, Hendrix S, et al. Clinically Meaningful Outcomes in Early Alzheimer Disease: A Consortia-Driven Approach to Identifying What Matters to Patients. Drug Inf J. 2017;51:380–390.

[5] Edgar CJ. Use of Qualitative Data to Support Content Validity of Performance-Based Cognitive Outcome Assessments. Therapeutic Innovation & Regulatory Science. 2017;51:671–671.

[6] Schoo LA, van den Berg E, van Zandvoort MJE, et al. Do You Know What I Mean? Laypeople and Experts’ Concepts of Cognition. Archives of Clinical Neuropsychology. 2012;27:182–189.

[7] Duffey MM, Ayuku D, Ayodo G, et al. Translation and Cultural Adaptation of NIH Toolbox Cognitive Tests into Swahili and Dholuo Languages for Use in Children in Western Kenya. Journal of the International Neuropsychological Society. undefined/ed;1–10.

[8] Boogert NJ, Madden JR, Morand-Ferron J, et al. Measuring and understanding individual differences in cognition. Philosophical Transactions of the Royal Society B: Biological Sciences. 2018;373:20170280.

[9] Dingwall KM, Pinkerton J, Lindeman MA. “People like numbers”: a descriptive study of cognitive assessment methods in clinical practice for Aboriginal Australians in the Northern Territory. BMC Psychiatry. 2013;13:1–13.

[10] Kearins JM. Visual spatial memory in Australian Aboriginal children of desert regions. Cognitive Psychology. 1981;13:434–460.

[11] Pietschnig J, Voracek M. One Century of Global IQ Gains: A Formal Meta-Analysis of the Flynn Effect (1909–2013). Perspect Psychol Sci. 2015;10:282–306.

[12] Chaytor N, Schmitter-Edgecombe M. The Ecological Validity of Neuropsychological Tests: A Review of the Literature on Everyday Cognitive Skills. Neuropsychology Review. 2003;13:181–197.

[13] Ruse SA, Davis VG, Atkins AS, et al. Development of a Virtual Reality Assessment of Everyday Living Skills. JoVE (Journal of Visualized Experiments). 2014;e51405.
